# BrachySound: machine learning based assessment of respiratory sounds in dogs

**DOI:** 10.1038/s41598-023-47308-0

**Published:** 2023-11-20

**Authors:** Ariel Oren, Jana D. Türkcü, Sebastian Meller, Teddy Lazebnik, Pia Wiegel, Rebekka Mach, Holger A. Volk, Anna Zamansky

**Affiliations:** 1 Information Systems Department, University of Haifa, Haifa, Israel; 2 Department of Small Animal Medicine and Surgery, University of Veterinary Medicine Hannover, Hannover, Germany; 3 Cancer Institute, University College London, London, UK; 4 Department of Mathematics, Ariel University, Ariel, Israel

**Keywords:** Animal behaviour, Zoology

## Abstract

The early and accurate diagnosis of brachycephalic obstructive airway syndrome (BOAS) in dogs is pivotal for effective treatment and enhanced canine well-being. Owners often do underestimate the severity of BOAS in their dogs. In addition, traditional diagnostic methods, which include pharyngolaryngeal auscultation, are often compromised by subjectivity, are time-intensive and depend on the veterinary surgeon’s experience. Hence, new fast, reliable assessment methods for BOAS are required. The aim of the current study was to use machine learning techniques to bridge this scientific gap. In this study, machine learning models were employed to objectively analyze 366 audio samples from 69 Pugs and 79 other brachycephalic breeds, recorded with an electronic stethoscope during a 15-min standardized exercise test. In classifying the BOAS test results as to whether the dog is affected or not, our models achieved a peak accuracy of 0.85, using subsets from the Pugs dataset. For predictions of the BOAS results from recordings at rest in Pugs and various brachycephalic breeds, accuracies of 0.68 and 0.65 were observed, respectively. Notably, the detection of laryngeal sounds achieved an F1 score of 0.80. These results highlight the potential of machine learning models to significantly streamline the examination process, offering a more objective assessment than traditional methods. This research indicates a turning point towards a data-driven, objective, and efficient approach in canine health assessment, fostering standardized and objective BOAS diagnostics.

## Introduction

The demand for brachycephalic (flat-faced) dog breeds has increased dramatically over the last few years^1^. As a result, the welfare concerns associated with extreme brachycephaly have entered the public consciousness^2–5^. In association with their conformation due to excessive breeding selection, primary abnormalities such as an oversized soft palate, stenotic nostrils, redundant pharyngeal folds, a hypoplastic trachea, obstruction of the nasal, pharyngeal, and laryngeal lumen, and other anatomic changes cause severe breed-related health problems^6–8^.

The Brachycephalic Obstructive Airway Syndrome (BOAS) is a chronic, pathophysiological disorder frequently observed in many short-muzzled breeds^5,7,8^. It leads to significant respiratory distress in affected individuals, including intolerance to heat and exercise, disrupted sleep patterns, cyanosis, and even collapses^9–12^.The excessive obstruction of the airways can lead to the manifestation of increased breathing noises such as stertor, stridor, and nasal sounds^7^. In severe cases, this condition can cause tracheal and laryngeal collapse in dogs as well as syncope, which can be fatal^13–15^.It is noticeable that brachycephalic breeds, due to such health issues, generally have a shortened life expectancy-averaging a reduction of 4.1 years^16^.

To reduce the advancement of secondary changes, an early diagnosis of BOAS is necessary as treatment and management can be started sooner. This has been shown to be critical in the clinical success rate^13,17,18^. The diagnostic significance of respiratory indicators in pets has received insufficient research attention^19,20^. A major challenge with BOAS is its frequent lack of recognition by the general public, as well as by veterinary surgeons^7,21–24^.Moreover, the lack of a standardized, objective and easily applicable method for diagnosing BOAS often leads to underdiagnosis, causing its prevalence and effects to be routinely underestimated^6,11,13,25^.

For instance, to assess the affectedness and severity of BOAS, Liu et al.^6^introduced and validated a functional grading system^6^. It is based on an assessment of respiratory clinical signs both before and after a 3-min exercise test. Potentially occurring breathing noises, the intensity of the respiratory effort, cyanosis or syncope are evaluated. To identify and classify breathing sounds, pharyngolaryngeal auscultation is performed by using a stethoscope. However, there is a certain subjectivity, especially in the grading process. This makes it challenging, even for veterinarians, to determine which dogs require a surgical intervention^5,21,26^

Aligned with other disciplines^27–31^, dogs’ behavioral analysis seems to move toward the adoption of modern computational and data-driven models such as machine learning and deep learning-based models^32–35^.Indeed, recent advances in machine learning have shown the potential for objective automated respiratory sound analysis to serve as a biometric tool for assessing respiratory function in humans^36,37^.In a similar vein, Oikarinen et al.^38^ utilized deep convolutional neural networks for the classification of marmoset vocalizations, highlighting the potential of such techniques in animal sound classification and source attribution. Xia et al.^39^ reviewed machine learning methods for audio-based respiratory condition screening, discussing also the use of machine learning for auscultation by the respiratory system. In the field of human medicine, machine learning algorithms have been developed for the detection of cardiac murmurs using digital stethoscope platforms, showcasing the versatility and potential of these techniques across various medical applications^40^. Contrary to the extensive research in human respiratory sound analysis, the use of machine learning in respiratory sound analysis in veterinary science remains underexplored. Grobman et al.^20^ collected acoustic recordings of respiratory and non-respiratory sounds in healthy dogs. The authors demonstrated that respiratory sounds could be reliably distinguished from other sounds using temporal and spectral descriptors. A study on horses also demonstrated the potential of spectral analysis of respiratory sounds for diagnosing upper airway disorders^41^.

### Study objectives

In this study, we used machine learning algorithms to analyze audio data collected with a digital stethoscope during a standardized fitness test. The aim was to diagnose BOAS and address the following objectives:

- **BOAS test result classification (Pass/Fail):** The classification of BOAS test results as either “pass” or “fail” based on respiratory sounds recorded after the fitness test.
- **BOAS test result prediction (Pass/Fail):** The prediction of BOAS test results (either “pass” or “fail”) using respiratory sounds recorded at rest, before initiating the fitness test.
- **Laryngeal sounds detection:** The detection of laryngeal sounds (stridor) within the respiratory sounds of dogs that have failed the BOAS test.

## Materials and methods

The research methodology consisted of two primary phases. Initially, a data collection process was undertaken, involving the recording of dogs with diverse BOAS grades in various brachycephalic breeds. Subsequently, leveraging the acquired dataset, a machine learning-based solution was developed and trained to address the two research inquiries investigated in this study. A comprehensive elucidation of each of these phases is presented below.

### Data collection

Data collection was performed at the Department of Small Animal Medicine & Surgery of the University of Veterinary Medicine, Hannover, Germany. The dogs’ owners provided written informed consent to provide data for this research, regulated by the law and regulations for research in Lower Saxony, Germany. All experiments were performed in accordance with relevant guidelines and regulations. The current protocol was reviewed and approved by an institutional Ethics Commission (Lower Saxony State Office for Consumer Protection and Food Safety (LAVES)), Oldenburg, Germany; the Ethical Committee of the University of Haifa waived ethical approval. The study population included 69 Pugs for Study 1, and 79 dogs of the breeds Affenpinscher, Boston Terrier, Brussels Griffon, French Bulldog, English Bulldog, Japanese Chin and Pekingese for Study 2. Inclusion criteria additional to their breed included: (i) age over 2 years, (ii) no acute illness, (iii) no lameness, (iv) no previous BOAS surgery, and (v) no respiratory distress at rest. All dogs were privately owned and acquired through social media, the German Kennel Association (Verband für das deutsche Hundewesen, VDH), and flyers. Breathing sounds were recorded within the context of two studies performed at the Department of Small Animal Medicine & Surgery of the University of Veterinary Medicine, Hannover, Germany. The first dataset included 214 audio samples of 69 Pugs, collected between July 2019 and August 2020, in a study evaluating a treadmill-based submaximal fitness test in Pugs^42^. Between August 2022 and May 2023, the second dataset of 152 samples was recorded as part of a follow-up study, including various brachycephalic breeds. In both studies, the dogs completed a 15-min fitness test, trotting at an individual comfort speed on a motorized treadmill. Prior to the test, pharyngolaryngeal auscultation was performed as part of a general examination, and breathing sounds were recorded using a 3M Littmann${\circledR }$ electronic stethoscope (model 3100BK27). In study 1, breathing noises were recorded and manually annotated at rest before (class ’Rest’) and after 5, 10, and 15 min during the fitness test (classes ’After5,’ ’After10,’ and ’After15’ respectively). In study 2, breathing noises were recorded and manually annotated before (class ’Rest’) and directly after the fitness test (class ’After’).

### Fitness test

The submaximal, standardised fitness test was evaluated for Pugs in 2022 at the Clinic of Small Animals of the University of Veterinary Medicine, Hannover, Germany^42^. The participants trotted at their individual comfort speed for 15 min on a treadmill in a room with controlled air conditions. Initially, a general examination was performed in which heart rate, respiratory rate, and temperature were determined. In addition, the weight was documented, and the body condition score was assessed using a scale of 1-9 points. Before the test started, breathing sounds were recorded with the electronic stethoscope. After 5 and 10 min, there was a break to measure and document heart rate and respiratory rate, and breathing sounds. The heart rate was permanently monitored during the testing period using a Polar heart rate sensor (Polar H1, sensor, and Polar FT7N, monitor, Polar Electro GmbH Deutschland, Büttelborn, Germany). The exercise was followed by a 15-min recovery period during which the dogs’ vital parameters should return to their initial values with a tolerance of 10%. Breathing noises were classified from grade 0-3 according to the BOAS grading by Liu et al.^6^. Dogs were considered to have passed the test if their vital parameters returned to baseline within the recovery period. In addition, they must present either no or only mild breathing noises, no moderately or severely increased respiratory effort or signs of dyspnoea or cyanosis, which corresponds to the initial two categories of the BOAS functional grading system. Dogs, that do not reach their initial vital parameters, show moderate to severe breathing noises, significantly increased respiratory effort or signs of dyspnoea or cyanosis have failed the test.

### Annotation of pharyngeal and laryngeal respiratory noises

The recorded respiratory noises were meticulously categorized by veterinarians into distinct classifications: absence of pathological breathing noises, and low, medium, or high-grade intensities of breathing noises. These sounds were further differentiated based on their periodicity, either as intermittent or constant. Moreover, a distinction was established among stertor, stridor, and nasal sounds. Stertor, characterized by its lower frequency and reminiscent of snoring, is often linked to nasal, pharyngeal, or tracheal pathologies^10^. Commonly, it is associated with conditions such as an elongated soft palate or obstructions in the nasopharyngeal region^18,19^.

Conversely, stridor presents as a high-frequency, whistling respiratory sound. Its etiology is attributed to turbulent airflow resulting from a constricted laryngeal aperture, narrowed laryngeal region, or instances of laryngeal collapse^18,19,21^. Nasal respiratory sounds, on the other hand, arise from pronounced stenosis of the nares and nasal passages, which is a prevalent condition in brachycephalic breeds^2^.

### Datasets preparation

- **BOAS test outcome. **
  - *Pugs*. Data were collected from 69 Pugs with a total of 214 recordings. These were categorized into four distinct phases of exertion: resting state, post 5 min, post 10 min, and post 15 min of physical activity. Specifically, the dataset included 70 samples recorded during the resting phase, 51 samples taken after 5 min of activity, 48 samples taken after 10 min of activity, and 45 samples captured immediately after the 15 min test. These pug dataset segments are denoted as Pugs$_{t}$ for $t\in \{rest, 5m,10m,15m\}$. Our primary objective was to identify BOAS-affected Pugs that only manifest clinical signs of impairment and altered breathing noises under exercise. To ensure representation from all exertion phases, we selected a subset of 136 recordings from 34 Pugs. Out of these, 17 Pugs passed the test while 17 did not, providing a balanced outcome for the dataset. Additionally, for consistency in analysis, all recordings were trimmed to a uniform length of 5.2 s to capture the Pugs’ breathing patterns consistently across different intervals.
  - *Various Breeds*. We also included data from 79 dogs of various brachycephalic breeds with 152 recordings. These recordings were performed only in two phases: at rest (before the test) and after 15 min of exercise. In detail, there were 82 recordings at rest and 70 recordings after exercise. These various breeds dataset segments are denoted as Var$_{t}$ for $t\in \{rest, 15m\}$. To ensure a balanced dataset, we curated subsets from these 152 recordings, aiming for equal representation from both the resting and post-exercise phases. Consequently, our dataset included 34 recordings from the resting phase and 34 recordings after 15 min of exercise. Similar to the Pug data, all recordings were trimmed to a consistent length of 5.2 s to ensure our analysis was based on standardized data segments.
- **Detection of laryngeal sounds (stridor)** For this phase, only the Pug-related data was used due to the limited amount of data available for various breeds and thus there was not enough data for the group of various breeds to conduct a well-powered statistically analysis. Out of the 69 Pugs, 28 failed the BOAS test, resulting in 98 recordings. Recordings in this dataset were shortened to 6 s, the shortest observed duration. This dataset is denoted as Laryng. During the resting phase, of the 27 dogs examined, 18 recordings predominantly showcased pharyngeal sounds, while the remaining 10 captured a mix of both pharyngeal and laryngeal sounds. After 5 min of exercise, the recordings of 16 of 23 dogs presented pharyngeal sounds, and the other eight displayed a combination of pharyngeal and laryngeal sounds. This distribution was consistent after 10 min of exercise in the same 23 dogs; most recordings showed pharyngeal sounds, with eight exhibited a mix of both types. Finally, following 15 min of exercise with 21 dogs, 18 recordings were distinctly pharyngeal breathing noises, whereas three integrated both pharyngeal and laryngeal sounds.

### Feature extraction and selection

We utilized the openSMILE library^43^ and its ComParE 2016^44,45^ feature set to pre-process the raw audio data. This library is renowned for its capability to extract an expansive set of 6373 features from audio data, encompassing attributes like signal energy, loudness, spectra, MFCC, pitch, and voice quality.

Given the high dimensionality of the extracted features, it was imperative to emphasize the most pertinent ones. Feature selection not only streamlines the dataset but can also enhance the performance of machine learning models. For this purpose, we employed the chi-squared test^46^. This test gauges the interdependence of stochastic variables, enabling the elimination of features that are likely independent of the classification task at hand. To determine the optimal number of features, we turned to cross-validation. By evaluating various feature counts, we identified the quantity that yielded the best cross-validation score, ensuring a data-driven decision rather than an arbitrary one. Post-selection, we applied min-max normalization to harmonize the scale of all features, a pivotal step since many algorithms are sensitive to feature scales. With the most salient features identified and scaled, we proceeded to train our machine learning models, aiming for enhanced performance and clearer interpretability.

### Machine learning models

All of the employed models share the same machine learning pipeline, utilizing a combination of K-Nearest Neighbors (KNN)^47^ and Decision Tree Classifier models^48^, integrated within a Majority Voting classifier. The ensemble approach was chosen to leverage the strengths of each individual model and to provide a more robust and reliable classification^49^.

Two configurations of the KNN model were employed. The first KNN model was designed to give weight to immediate neighbors, capturing local patterns in the data. The second KNN model was configured to provide a more global perspective, capturing broader patterns across the dataset. This dual approach was based on the hypothesis that similar behaviors among pugs would be effectively captured at both local and global levels. The Decision Tree Classifier was used as an extrapolator to capture more complex, non-linear relationships in the data. The Majority Voting Classifier was then used to combine the predictions of the two KNN models and the Decision Tree Classifier. This approach ensures that the final prediction is not overly reliant on any single model.

As we had two different datasets (Pugs and various breeds), we could investigate the generalizability of the approach across these groups of breeds. Thus, in the BOAS test result tasks, we experimented with different combinations for training/testing sets: pugs/pugs, various/various, pugs/various, various/pugs and pugs+various/pugs+various.

The *leave-one-out* cross-validation method was employed^50,51^. In this method, if there are $N$ data points in the dataset, the model is trained $N$ times. Each time, the model is trained using $N-1$ data points and tested on the one left out. This is repeated until each data point has been used as the test set once.

The developed machine models addressed the following three tasks:

1. Classification of BOAS test results (pass/fail): The models were trained and tested on data from the following datasets:
  Pugs at 5 min post-exercise ($\textsf {Pugs}{5}$)
  Pugs at 10 min post-exercise ($\textsf {Pugs}{10}$)
  Pugs at 15 min post-exercise ($\textsf {Pugs}{15}$)
  Various breeds at 15 min post-exercise ($\textsf {Var}{15}$)
2. Prediction of BOAS test results (pass/fail): The models were trained and tested on data from the following datasets:
  Remaining Pugs data ($\textsf {Pugs}{rest}$)
  Remaining Various breeds data ($\textsf {Var}{rest}$)
3. Detection of laryngeal sounds (present/absent): The models were trained and tested on the following dataset:
  Laryngeal sounds from Pugs ($\textsf {Laryng}$)

Parameters for each model have been optimized using the grid search method^52^ to maximize the performance, specifically using the F1 score. for the KNN models, we explored configurations with 3 or 5 neighbors using a distance weight and either an Euclidean or Manhattan metric and another set with 7 or 9 neighbors with both uniform and distance weights. For the Decision Tree Classifier, we varied the maximum depth from 2 to 5 and tested both the Gini and Entropy criteria^53^.

**Figure 1:**
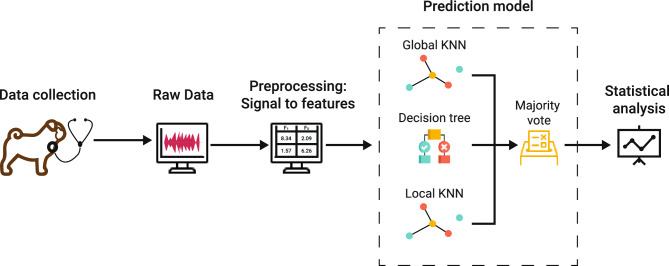
Schematic representation of the ensemble model architecture for predicting BOAS affectedness in brachycephalic dogs. Raw data undergoes a condensed preprocessing pipeline, including feature extraction with the openSMILE library and normalization. The primary focus of the architecture is the employment of two distinct KNN configurations: one accentuating immediate neighbors for local patterns and another capturing broader dataset patterns. These are complemented by a Decision Tree Classifier designed to unravel complex, non-linear relationships. A Majority Voting Classifier amalgamates the predictions from each model to produce a balanced final output, ensuring no undue emphasis on any single model.

Figure 1 summarizes the pipeline.

### Feature importance

Permutation importance^54^ is a method to determine feature significance in a machine learning model. The approach works by randomly shuffling a single feature and measuring the decrease in model performance. Features that are crucial to the model’s decision-making process will, when permuted, lead to a significant drop in model performance. In contrast, less relevant features will result in a smaller decrease.

In this study, permutation importance was employed post-hoc after training the Majority Voting Classifier. This was to ensure that the significance of features was consistent with the model’s decision-making process. In order to provide a safeguard against potential overfitting or spurious associations, a noise column was incorporated. Features that demonstrated an importance score comparable to, or lower than the noise column, were earmarked as less-informative or non-contributive.

Importance scores were aggregated across all iterations, providing an average importance value for each feature. These values were used to rank the features in terms of their relevance to the model’s decision-making process.

## Results

**Classification of BOAS test results (pass/fail)** The performance of the classification models trained on different dataset segments are presented in Table 1. Combining samples taken after 5 min, 10 min, and 15 min of exercise gives the best accuracy of 85% for Pugs. The model for Pugs generalizes well for the group of various brachycephalic breeds, reaching an accuracy of 71%. Training on various breeds and testing on Pugs, however, yields a worse performance of only 68%, the same as training on the group of various breeds and testing on this group.

**Table 1:** Performance metrics of the BOAS test outcome classifier model across different breed datasets: ${\textsf {Pugs}}$ and ${\textsf {Various}}$.

Trained On	Tested On	Segment	Accuracy	Precision	Recall	$F_1$
Pugs	Pugs	${5m}$	0.82	0.84	0.82	0.82
		${10m}$	0.65	0.66	0.65	0.64
		${15m}$	0.79	0.80	0.79	0.79
		$5m + {10m}$	0.82	0.83	0.82	0.82
		$5m + {15m}$	0.74	0.74	0.74	0.74
		${10m} + {15m}$	0.79	0.80	0.79	0.79
		$5m + {10m} + {15m}$	**0.85**	**0.86**	**0.85**	**0.85**
Various	Various	${15m}$	0.68	0.68	0.68	0.68
Pugs	Various	${15m}$	0.71	0.81	0.71	0.68
Various	Pugs	${15m}$	0.68	0.71	0.68	0.66

Metrics including accuracy, precision, recall, and $F_1$ score are presented for various training and testing scenarios and data segments. The best results are highlighted in bold font.

**Figure 2:**
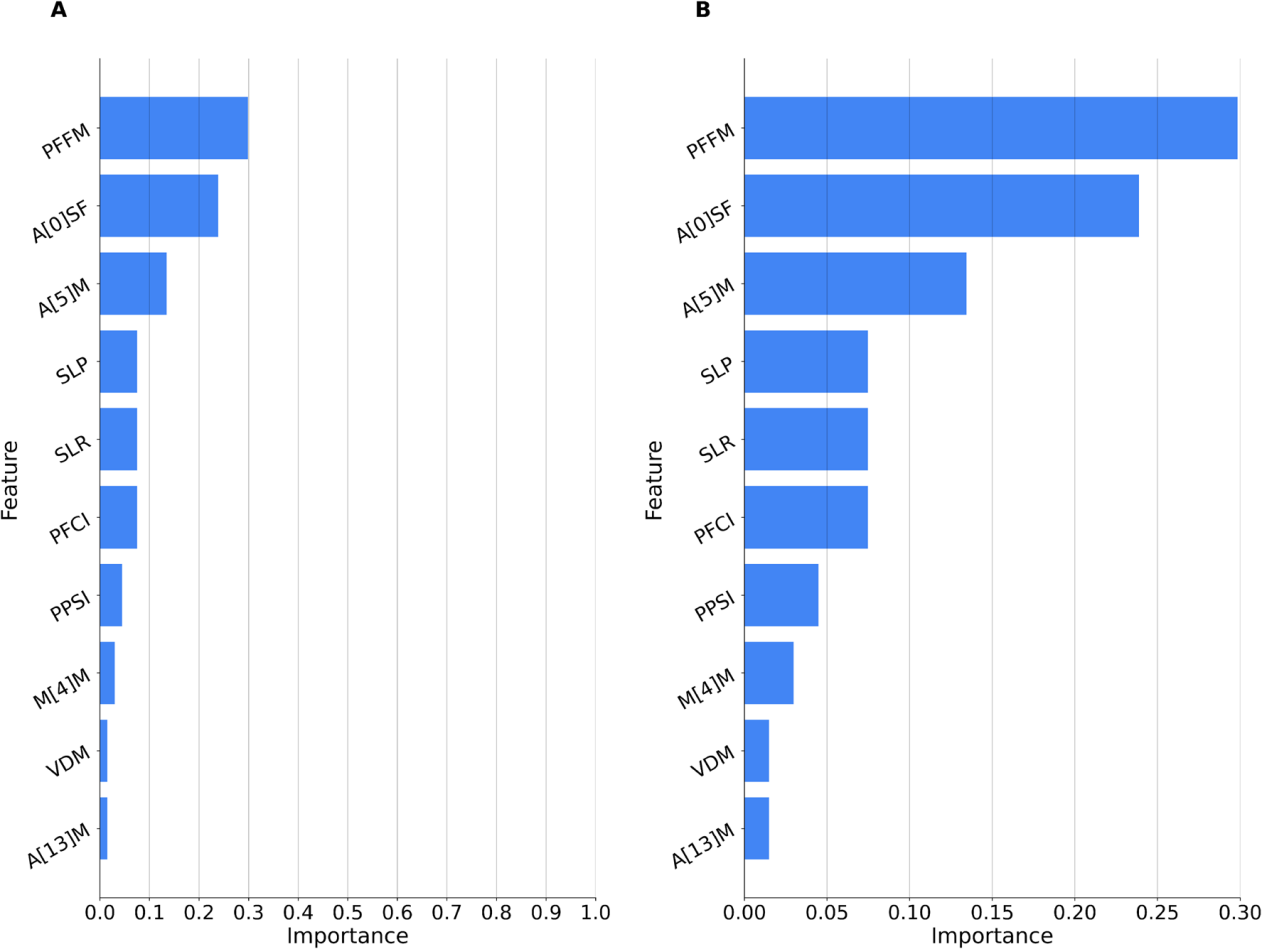
Feature importance for predicting BOAS outcome based on respiratory sounds at rest. The model trained on ${\textsf {Pugs}}_{rest}$ dataset is tested on ${\textsf {Var}}_{rest}$ dataset. The graph is divided into two sections, A and B, with B being identical to A but with the Y-axis scaled to [0, 0.3] to better show the differences between the features. The features, in order of importance, include: **PFFM** (Spectral flux minimum segment length), **A[0]SF** (0th auditory spectral filter standard deviation of falling slope), **A[5]M** (5th auditory spectral filter minimum segment length), **PFCI** (Spectral centroid interquartile range), **SLP** (Local shimmer percentile range 0-1), **SLR** (Local shimmer range), **PPSI** (Psychoacoustic sharpness interquartile range), **M[4]M** (4th Mel-frequency cepstral coefficient minimum segment length), **VDM** (Voicing final unclipped minimum position), and **A[13]M** (13th auditory spectral filter minimum segment length). This illustrates the relevance of specific audio features in prediction accuracy across different brachycephalic breeds.

**Figure 3:**
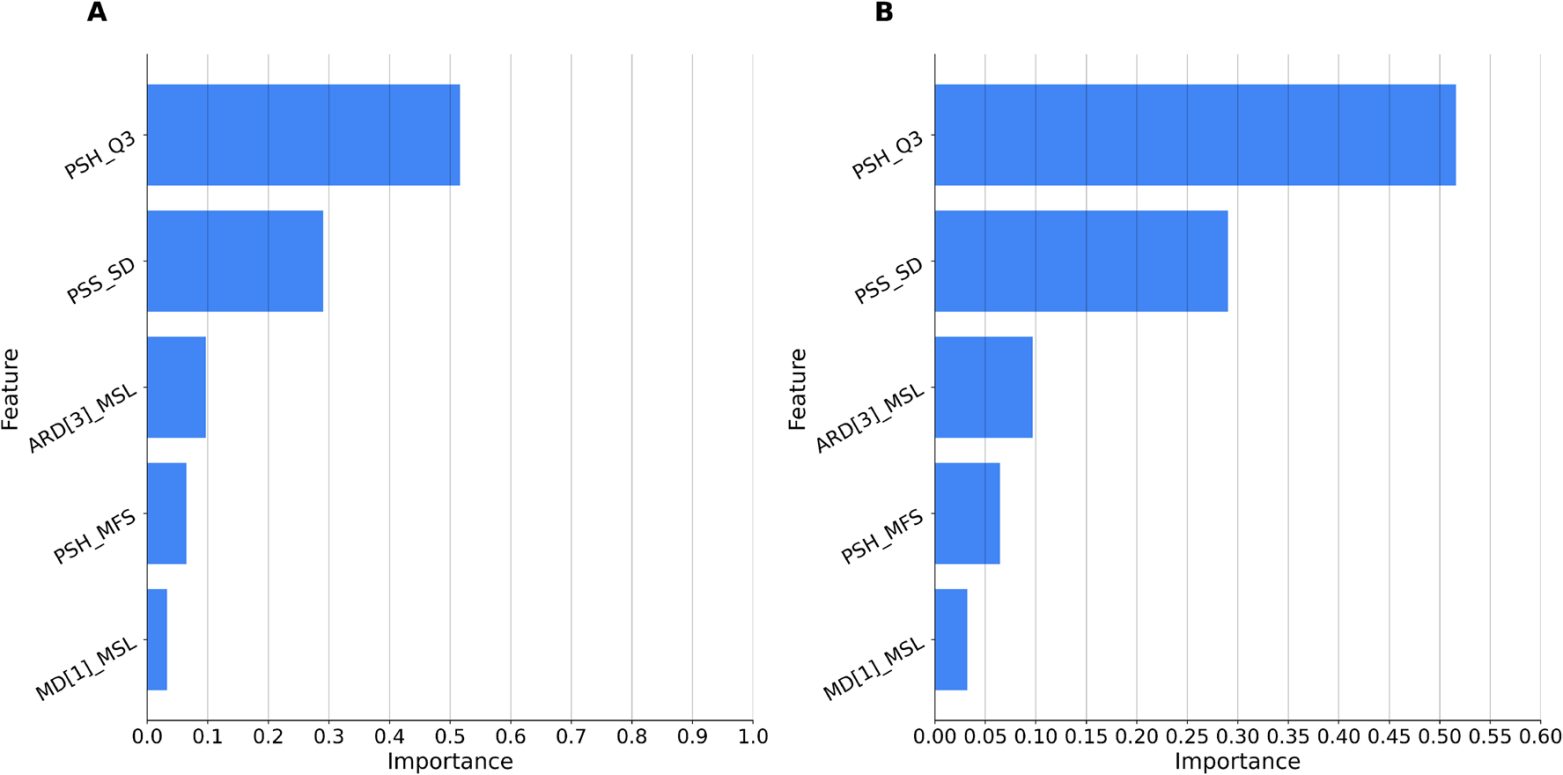
Feature importance for predicting BOAS outcome based on respiratory sounds at rest. The model, trained on the ${\textsf {Var}}_{rest}$ dataset and tested on the ${\textsf {Pugs}}_{rest}$ dataset, underscores the significance of specific audio features in achieving prediction accuracy. The graph is divided into two sections, A and B, with B being identical to A but with the Y-axis scaled to [0, 0.6] to better show the differences between the features. The features, ranked by their normalized importance, encompass: **PSH**_**Q3** (Spectral harmonicity third quartile), **PSS**_**SD** (Spectral slope standard deviation), **ARD[3]**_**MSL** (3rd auditory spectral filter derivative minimum segment length), **PSH**_**MFS** (Spectral harmonicity mean falling slope), and **MD[1]**_**MSL** (1st MFCC derivative minimum segment length). These features highlight the relevance and contribution of each audio attribute in the prediction process, providing insights into their significance in the model’s performance.

**Prediction of BOAS test results (pass/fail)** Table 2 shows the performance of prediction models for BOAS test results for different scenarios of training and testing using the rest segment of the datasets. Models trained on Pugs and tested on Pugs and various breeds reach an accuracy of 68%. Figures 2 and 3 show feature importance for the best model (highlighted in **bold**).

**Table 2:** Classifier performance in predicting the BOAS outcome across different breed datasets: ${\textsf {Pugs}}$ and ${\textsf {Various}}$.

Trained On	Tested On	Segment	Accuracy	Precision	Recall	$F_1$
Pugs	Pugs	Rest	0.68	0.68	0.68	0.67
Various	Various	Rest	0.65	0.65	0.65	0.65
Pugs	Various	Rest	**0.68**	**0.68**	**0.68**	**0.68**
Various	Pugs	Rest	0.65	0.65	0.65	0.65

Metrics including accuracy, precision, recall, and $F_1$ score are presented for various training and testing combinations. The best results are highlighted in bold font.

**Figure 4:**
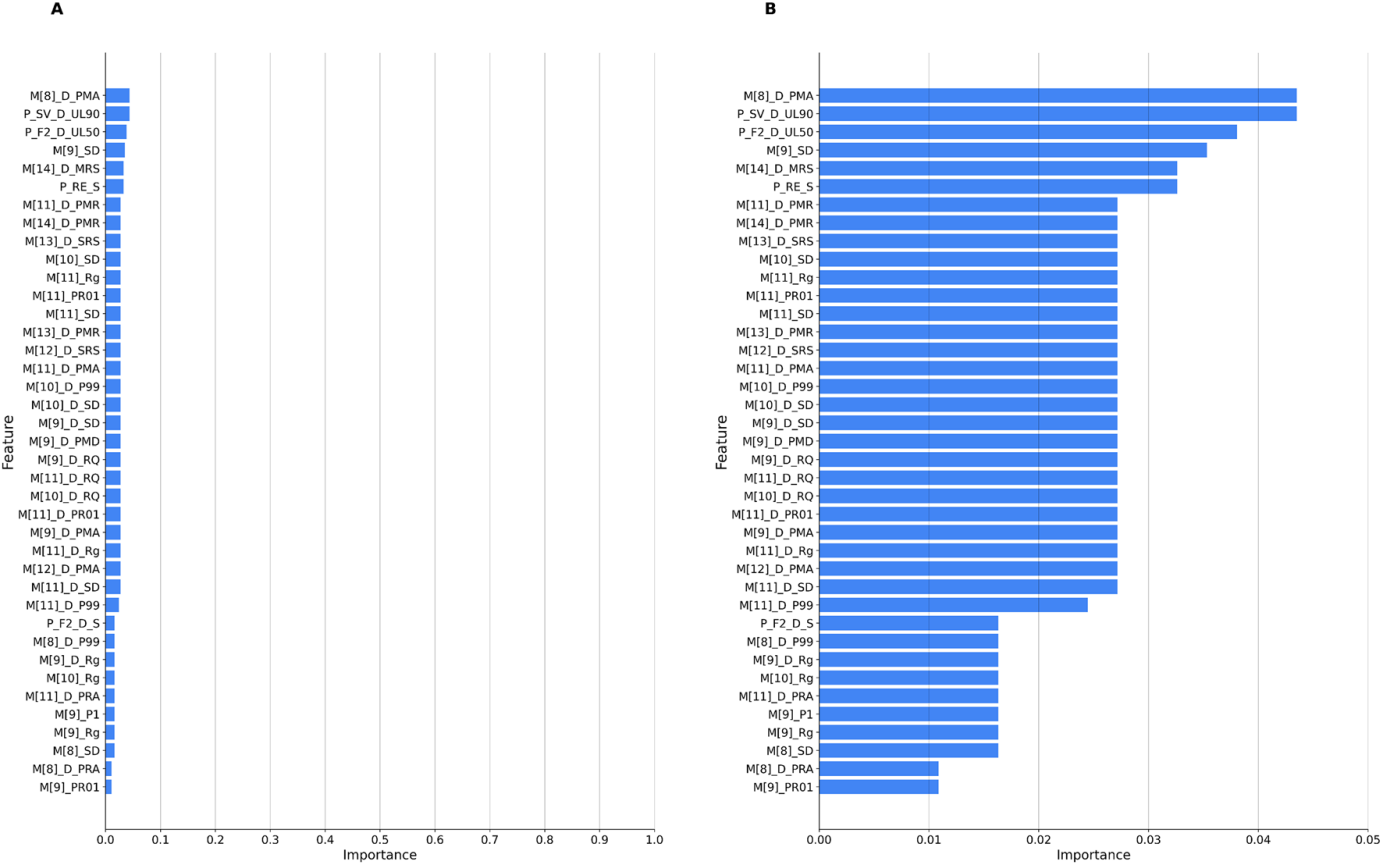
Feature importance for detecting laryngeal sounds presence or absence. Graph A represents the full range of feature importances, while Graph B is identical to A but with the Y-axis scaled to [0, 0.05] to better show the differences between the features. The model, trained and tested on ($\textsf {Laryng}$), underscores the significance of specific audio features in accurately distinguishing these sounds. The features, in descending order of importance, include: **P**_**SV**_**D**_**UL90** (Spectral variance derivative uplevel time 90%), **M[8]**_**D**_**PMA** (8th MFCC derivative peak mean absolute), **P**_**F2**_**D**_**UL50** (Spectral energy in the 250-650 Hz band derivative uplevel time 50%), **M[14]**_**D**_**MRS** (14th MFCC derivative mean rising slope), **P**_**RE**_**S** (RMS energy minimum segment length), **M[9]**_**SD** (9th MFCC standard deviation), **M[10]**_**SD** (10th MFCC standard deviation), **M[11]**_**SD** (11th MFCC standard deviation), **M[9]**_**D**_**SD** (9th MFCC derivative standard deviation), and **M[10]**_**D**_**SD** (10th MFCC derivative standard deviation). This comprehensive list provides insights into the relevance and significance of each audio feature in the detection process.

**Laryngeal sounds detection (present/absent)** Table 3 shows the classifier’s performance metrics for the ${\textsf {Laryng}}$ dataset, reaching an accuracy of 83%. Fig. 4 shows feature importance.

**Table 3:** Performance metrics of the classifier for detecting laryngeal sounds.

Dataset	Accuracy	Precision	Recall	$F_1$ score
Laryng	**0.83**	**0.83**	**0.79**	**0.80**

Metrics including accuracy, precision, recall, and $F_1$ score.

## Discussion

This study explored machine learning techniques for objective analysis of brachycephalic dogs’ respiratory sounds. To address this study objective a dataset of audio data obtained via digital stethoscope during a standardized BOAS fitness test was employed. Models for classifying BOAS test results based on audio obtained during the exercise test reached 85% accuracy for the Pug dataset and generalized well to other brachycephalic breeds, reaching accuracy of 71% for previously unseen data of the other group of dogs. The use of this algorithm revealed that a combination of samples after 5 min, 10 min and 15 min showed the highest accuracy, precision and thus an F1-score of 0.85. For the group of various brachycephalic breeds, it was only possible to program an algorithm based on recordings after 15 min of exercise due to the limited data availability. This showed a considerably lower performance in comparison with an F1 score of 0.68. Therefore, it seems to be necessary to collect and analyze more audio samples recorded during exercise in order to develop a more precise and accurate algorithm. The obtained model for predicting BOAS test results based on audio data recorded at rest, before the exercise test started, reached 68% accuracy for the algorithm trained on Pugs. For both groups, the algorithm was based on audio data recorded exclusively at one time point, allowing a better comparison of the performances between them. With an F1 value of 0.67 for Pugs and a value of 0.65 for dogs of different brachycephalic breeds, a quite similar result was obtained. Comparing the performance of all algorithms, it can again be observed that the highest accuracy could still be achieved with the combination of the recordings during exercise of the three time points (5 min, 10 min, 15 min). This shows that the exercise within the BOAS diagnostics is crucial to identify affected dogs as accurately as possible. Finally, the model for the detection of laryngeal sounds reached accuracy of 83%, which is a strong result despite the limited availability of samples. The superior performance of our machine learning model, compared to the widely-used XGBoost^55^, highlights the potential benefits of tailoring algorithms specifically for veterinary diagnostic applications. Notably, the model demonstrated commendable cross-breed generalizability, with training on the Pugs dataset resulting in accurate predictions for the various brachycephalic breeds dataset and vice versa. This suggests overarching audio characteristics shared among brachycephalic breeds, which may be pivotal in diagnosing BOAS. The model’s success in predicting test results even during the resting phase further demonstrates its practical applicability in supporting diagnostics. However, there were inherent challenges. The lack of samples, especially for detecting laryngeal sounds within various brachycephalic breeds, poses a potential limitation. This data deficiency could potentially challenge the broad adaptability of our model to diverse breeds and respiratory conditions. Further investigation of breed-specific audio characteristics would be beneficial, since BOAS seems to have a different prevalence depending on the breed and expression of pathological changes in the upper airways may also differ^56,57^. It would therefore be useful to collect more recordings from different brachycephalic breeds from all test time points to be able to train the model further. Thus, it might be possible to identify specific differences in the breathing noises of different breeds and use them for diagnostic purposes as well. Furthermore, it would be possible to develop an algorithm that classifies the intensity of the breathing sounds according to the BOAS grading used in the fitness test. Pharyngeal breathing noises in particular are most common in many brachycephalic dogs, but even here it is difficult to objectively assess the intensity of these sounds. A machine learning algorithm would therefore be useful in assisting veterinary surgeons in their decisions. Nevertheless, in order to create the algorithm, it is necessary to classify the breathing noises into their different grades beforehand, which in turn poses the problem of subjectivity. Although there was not enough data available in this study for the purpose of classifying the different grades, all breathing noises were classified by several different veterinarians to address this issue. Additionally, another limitation arises from the controlled environment in which the respiratory sound recordings were conducted. These controlled settings do not fully represent real environments in clinical practice. Various factors like background noise, the dog’s activity level, and other environmental stressors could potentially affect the recordings and, consequently, the model’s predictive power. In addition, noises and thus also the background noises can be increased by the use of an electronic stethoscope. Recording breathing sounds on dogs that move a lot during recording can be difficult and the model must be trained not to identify them as breathing noise. It is important to have the application evaluated in the next step in the clinical environment by different veterinary surgeons and to adapt the algorithms to any new challenges that may arise. This enables the algorithm to further optimize the performance of the classification and predictions. In conclusion, while this study provides a potential way to support and improve conventional diagnostic methodologies and presenting an objective avenue for early detection of BOAS signs, it emphasizes the need for broader and more diverse datasets to ensure reliable predictions. Of course, it is necessary to consider that BOAS can present with a wide range of clinical signs, which is why the detection of breathing sounds is a very important aspect, but not the only factor. Still, the use of this algorithm in combination with a standardized exercise test and functional grading is a promising way to improve and objectively support early diagnosis. The findings illuminate both the immense potential and existing challenges of integrating machine learning into veterinary diagnostics.

## Data Availability

The datasets used and/or analysed during the current study available from the corresponding author on reasonable request.

## Feature descriptions

Table 4 summarizes the names and descriptions of the features used as part of the proposed model.

**Table 4:** Detailed Descriptions of Features and Their Short Names used for detecting the presence or absence of laryngeal sounds.

Feature description	Short name
Root mean square (RMS) energy minimum segment length	P_RE_S
8th Mel-frequency cepstral coefficient (MFCC) standard deviation	M[8]_SD
9th MFCC range	M[9]_Rg
9th MFCC 1st percentile	M[9]_P1
9th MFCC percentile range (0-1)	M[9]_PR01
9th MFCC standard deviation	M[9]_SD
10th MFCC range	M[10]_Rg
10th MFCC standard deviation	M[10]_SD
11th MFCC range	M[11]_Rg
11th MFCC percentile range (0-1)	M[11]_PR01
11th MFCC standard deviation	M[11]_SD
Spectral energy in the 250-650 Hz band derivative minimum segment length	P_F2_D_S
Spectral energy in the 250-650 Hz band derivative uplevel time 50%	P_F2_D_UL50
Spectral variance derivative uplevel time 90%	P_SV_D_UL90
8th MFCC derivative 99th percentile	M[8]_D_P99
9th MFCC derivative range	M[9]_D_Rg
9th MFCC derivative standard deviation	M[9]_D_SD
10th MFCC derivative 99th percentile	M[10]_D_P99
10th MFCC derivative standard deviation	M[10]_D_SD
11th MFCC derivative range	M[11]_D_Rg
11th MFCC derivative 99th percentile	M[11]_D_P99
11th MFCC derivative percentile range (0-1)	M[11]_D_PR01
11th MFCC derivative standard deviation	M[11]_D_SD
8th MFCC derivative peak range absolute	M[8]_D_PRA
8th MFCC derivative peak mean absolute	M[8]_D_PMA
9th MFCC derivative root quadratic mean	M[9]_D_RQ
9th MFCC derivative peak mean absolute	M[9]_D_PMA
9th MFCC derivative peak mean distance	M[9]_D_PMD
10th MFCC derivative root quadratic mean	M[10]_D_RQ
11th MFCC derivative root quadratic mean	M[11]_D_RQ
11th MFCC derivative peak range absolute	M[11]_D_PRA
11th MFCC derivative peak mean absolute	M[11]_D_PMA
11th MFCC derivative peak mean relative	M[11]_D_PMR
12th MFCC derivative peak mean absolute	M[12]_D_PMA
12th MFCC derivative standard deviation of rising slope	M[12]_D_SRS
13th MFCC derivative peak mean relative	M[13]_D_PMR
13th MFCC derivative standard deviation of rising slope	M[13]_D_SRS
14th MFCC derivative peak mean relative	M[14]_D_PMR
14th MFCC derivative mean rising slope	M[14]_D_MRS

The model, trained and tested on ($\textsf {Laryng}$), underscores the significance of these specific audio features in accurately distinguishing these sounds.
